# National trends in ideal cardiovascular health among adults in Bhutan from three cross-sectional surveys in 2007, 2014, and 2019

**DOI:** 10.1038/s41598-022-09688-7

**Published:** 2022-04-05

**Authors:** Supa Pengpid, Karl Peltzer

**Affiliations:** 1 Department of Health Education and Behavioral Sciences, Faculty of Public, Health Mahidol University, Bangkok, Thailand; 2 Department of Research Administration and Development, University of Limpopo, Polokwane, South Africa; 3 Department of Psychology, College of Medical and Health Science, Asia University, Taichung, Taiwan

**Keywords:** Cardiology, Diseases

## Abstract

The study aimed to estimate the prevalence, distribution, and correlates of ideal cardiovascular health (CVH) among individuals (20–69 years) across three cross-sectional surveys in 2007, 2014 and 2019 in Bhutan. Cross-sectional data were analysed from 9712 individuals (20–69 years, mean age = 37.6 years) who participated in the 2007, 2014 or 2019 Bhutan STEPS surveys, had complete measurement of CVH metrics, and had no history of a cardiovascular disease in 2014 and 2019. Ideal CVH measures included non-smoking, healthy diet, physical activity, body mass index (< 23 kg/m^2^), blood pressure < 120/ < 80 mmHg, total cholesterol < 200 mg/dL, and fasting blood glucose < 100 mg/dL). The prevalence of five to seven ideal CVH metrics increased from 11.6% in 2007 to 37.9% in 2019. Trend analyses showed that ideal physical activity, ideal total cholesterol, ideal blood pressure, and ideal fasting glucose increased from 2007 to 2019, while ideal fruit and vegetable intake, ideal smoking, and ideal body mass index decreased from 2007 to 2019. Five to seven ideal CVH metrics, 0–7 ideal CVH metrics, and 0–14 poor, intermediate, and ideal CVH metrics increased from 2007 to 2019. In the adjusted logistic regression analysis, older age decreased the odds of having 5–7 ideal CVH metrics in all three study years. Male sex increased the odds, and employment decreased the odds of 5–7 ideal CVH metrics in 2007, and urban residence increased the odds of 5–7 ideal CVH metrics in 2014 and decreased the odds in 2019. The proportion of meeting 5–7 ideal CVH metrics increased in Bhutan. Primary and secondary prevention programmes must be strengthened to improve CVH in Bhutan, considering identified associated factors.

## Introduction

Globally, an “estimated 17.9 million people died from cardiovascular diseases (CVDs) in 2019, representing 32% of all global deaths”, of which more than three-quarters occurred in low- and middle-income countries^1^. CVDs contribute to 28% of all death in 2016 in Bhutan, a lower middle-income country in south central Asia^2^. “Non-communicable diseases (56%) and injuries (a staggering 19%) account for over three-quarters of all deaths in Bhutan and the probability of dying prematurely (before the age of 70) from one of the four main NCDs is one in five”^3^. CVD risk factors in Bhutan include a “now high prevalence of overweight and obesity being observed among adult populations (i.e., 27% in men and 40% in women), two thirds of the population do not consume sufficient fruit and vegetables and smoke, and smokeless tobacco is widely used (34% in men and 14% in women)”^3^. These CVD risk factors often cluster together, increasing the risk of developing CVDs, and should be prioritized in the prevention of CVDs^4,5^.

In an effort to prevent the development of CVDs, the American Heart Association (AHA) conceptualized “ideal cardiovascular health (CVH)”, including seven ideal health factors and behaviours, including blood pressure, blood glucose level, total cholesterol, level of smoking, physical activity, body mass index, and nutritional intake^6,7^. Using these seven metrics, the CVH status of the population of the population can be defined as ideal (5–7 ideal metrics), intermediate (3–4 ideal metrics) or poor (0–2 ideal metrics)^8^. Having a higher number of ideal CVH metrics has been shown to be protective against “the risk of all-cause and CVD-related mortality, incident cardiovascular events, lower prevalence and incidence of non-CVD outcomes such as cancer, depression, and cognitive impairment”^9^. In more recent studies, for example, among a middle-aged Finnish population^10^ and a Chinese adult population, the CVH score decreased the risk of CVD mortality^11^. We could not find any reports on ideal CVH in Bhutan.

Globally, mainly in high-income countries, 19.6% of participants had ideal (5–7 ideal metrics) CVH^8^. Few reports have been found on CVH in East and South Asian low- and middle-income countries. Several studies in China found, e.g., in Shandong (18–69 years) 0.05% had all 7-ideal metrics^12^, in rural Northwest China (20–80 years) 0.0%^13^ and in rural China (≥ 35 years), 0.1% had all 7 ideal CVH metrics^14^. In a nationally representative sample in China (≥ 20 years), 33.0% had 5–7 ideal CVH^15^. In South Asia, in Nepal (15–69 years), 51.6% had 5–7 ideal CVH metrics^16^, in semi-urban Western Nepal (≥ 25 years), 14.3% had 6 or 7 ideal metrics^5^, and in urban India (20–75 years), ideal < 0.1% had 7 ideal metrics and 7.1% had ≥ 6 ideal metrics^17^. Globally, dietary pattern (12.1%) had the lowest prevalence of CVH status, followed by blood pressure (BP) (34.6%), body mass index (BMI) (40.3%), physical activity (40.6%), total cholesterol (TC) (51.7%), fasting blood glucose (FPG) (67.7%), and smoking (69.1%)^8^.

We could not find any trend study on ideal CVH in East and South Asian countries, with the exception of a study in North China, which however, does not report the total ideal CVH metrics^18^. In a trend study over ten years in Iran, the prevalence of 5–7 ideal CVH metrics slightly reduced from 22.7% in 2007 to 19.5% in 2016, the mean CVH metrics increased from 4.7 in 2007 to 5.0 in 2016^19^. In a trend study among adults in USA over seven years the prevalence of the ideal CVH score significantly increased from 3.89 in 2011 to 3.96 in 2017^20^, and a trend study over 20 years in France found a decrease in ideal CVH^21^.

Sociodemographic factors associated with ideal CVH may include female sex^8,16,22^, younger age^8,16,17,22,23^, ethnicity^22^, higher education^12,22,23^, and rural residence^24,25^. The study aimed to estimate the prevalence and associated factors of ideal CVH among individuals (≥ 20 years) in three cross-sectional surveys in 2007, 2014 and 2019 in Bhutan.

## Methods

### Study design and participants

Secondary data from three cross-sectional STEPwise approach to surveillance (STEPS) surveys in Bhutan in 2007, 2014, and 2019^26^ with complete CVH metrics measurements and no history of CVD in 2014 and 2019 were analyzed; the overall response rate was > 93% in 2014 > 96% in 2019^27–29^. “A multi-stage stratified sampling process was carried out to randomly select participants from the target population. An individual within the age range of the survey” was selected per household (25–74 years in 2007, 18–69 in 2014, and 15–69 years in 2019)^27–29^. In 2007 the study was restricted to the capital city of Thimphu, and in 2014 and 2019 the whole country of Bhutan. We restricted our data analyses to persons 20–69 years and those with no missing data on “smoking status, BMI, PA, diet, total TC, FBG and BP” measurements. The Research Ethics Board for Health (REBH), Bhutan, approved the study and written informed consent was obtained from all participants^27–29^. All methods were performed in accordance with the relevant guidelines and regulations.

Data collection followed the “WHO three STEPS methodology: step 1 included administration of a structured questionnaire (sociodemographics, medical history, medication use, and health risk behaviour) step 2 consisted of blood pressure and anthropometric measurements, and step 3 included biochemical tests (blood glucose and blood lipids)”^26^. Anthropometric measurements were taken with a portable digital weighing scale (SECA 843), constant tension Myotape tape (SecaTMbrand)^27–29^. Prior to taking blood pressure measurements, participants were asked to sit quietly and rest for 15 min with legs uncrossed. Three readings of systolic and diastolic blood pressure were obtained, with participants resting for three minutes between each reading. Of the three blood pressure measurements using “Omron BP apparatus automatic blood pressure monitor”^27–29^; the last two readings following recommendations by WHO were averaged^26^. “Blood glucose, and total cholesterol were measured in peripheral (capillary) blood at the data collection site using dry chemical methods, biochemical analysis with a Hitachi 912 bio-analyzer”^27–29^.

## Measures

Poor, intermediate and ideal CVH levels for “smoking, BMI, PA, diet, TC, BP, and FBG” were evaluated following modified AHA definitions, exact AHA classifications are given in brackets^6,7^.

### Cardiovascular health behaviour

*Smoking status:* ≥ 20 years, smoking is defined as poor if current smoker (in the past 12 months), and ideal if self-report not past 12-month (current) smoker (adults ≥ 20 years, intermediate, former smoker ≤ 12 months).

*Body Mass Index* (BMI) (kg/m^2^): “ ≥ 20 years, BMI is defined poor if ≥ 25 kg/m^2^, intermediate as 23.0–24.9 kg/m^2^, and ideal BMI is < 23.0 kg/m^2^”^30^ (“adults ≥ 20 years BMI is defined poor if ≥ 30 kg/m^2^, intermediate as 25.0–29.9 kg/m^2^, and ideal BMI is < 25 kg/m^2^”).

*Healthy diet:* adults ≥ 20 years, poor healthy diet is defined as “ < 2 servings of fruit and vegetables (FV)/day, intermediate as 2–< 4.5 FV/day, and an ideal diet as ≥ 4.5 FV servings/day”^31^; [adults ≥ 20 years, “poor: 0–1 components, intermediate: 2–3, and ideal: 4–5 components (1: ≥ 4.5 cups/day fruits and vegetables, 2: ≥ 3.5 oz servings/week of fish, 3: < 1500 mg/day sodium, 4: < 450 cal/week sweets/sugar, and 5: ≥ 3 1-oz servings/day whole grains)”].

*Physical activity* (PA): adults ≥ 20 years, “Poor = None, Intermediate =  < 600 MET mins/week, 600–< 1500 MET mins/week, and ≥ 1500 MET mins/week, based on the Global Physical Activity questionnaire^19,32^. (Adults ≥ 20 years, “Poor = None, Intermediate = 1–149 min/wk moderate intensity or 1–74 min/wk vigorous intensity or 1–149 min/wk moderate + vigorous, ideal =  ≥ 150 min/wk moderate intensity or ≥ 75 min/wk vigorous intensity or ≥ 150 min/wk moderate + vigorous”).

### Cardiovascular health factors

*Poor total cholesterol* (TC) is classified as adults ≥ 20 years, “poor is TC ≥ 6.3 mmol/L (≥ 240 mg/dL), intermediate is TC 5.2–6.2 mmol/L (200–239 mg/dL), and ideal TC is < 200 mg/dL” [adults ≥ 20 years, “intermediate is TC 5.2–6.2 mmol/L (200–239 mg/dL) or treated to TC < 5.2 mmol/L (< 200 mg/dL) and ideal TC is < 200 mg/dL and without any cholesterol-lowering medication”].

*Fasting blood glucose (FBG)*: adults ≥ 20 years, poor FBG is defined as “glucose ≥ 7.0 mmol/L (≥ 126 mg/dL), intermediate is glucose 5.6–6.9 mmol/L (100–125 mg/dL) or treated to < 100 mg/dL, and ideal is < 5.6 mmol/L < 100 mg/dL and without any glucose-lowering medication” [Adults ≥ 20 years].

*Blood pressure* (BP): adults ≥ 20 years, poor is defined as “BP ≥ 140/ ≥ 90 mmHg, intermediate is systolic BP 120–139 mmHg or diastolic BP 80–89 mmHg or treated to BP < 120/ < 80 mmHg, and ideal BP is defined as BP < 120/ < 80 mmHg and without any antihypertensive medication” [Adults ≥ 20 years].

The seven CVH metrics were coded as 1 = ideal and 0 = not ideal, summed, and classified into 0–2, 3–4, and 5–7 ideal CVH metrics.

In STEPS surveys 2014 and 2019 but not in 2007, “history of CVDs included self-reported coronary heart disease; angina, also called angina pectoris; a heart attack (also called myocardial infarction); stroke; any kind of heart condition or heart disease (other than the ones I just asked about) (Yes, No)”^26^.

Sociodemographic covariates included age (years), sex (male, female), education in years, number of adult household members, employment, and residence status^24^.

### Data analysis

All statistical analyses were conducted with “STATA software version 14.0 (Stata Corporation, College Station, TX, USA)”. “Analysis weights were calculated by taking the inverse of the probability of selection of each participant. These weights were adjusted for differences in the age-sex composition of the sample population as compared to the target population”. Taylor linearization methods were utilized to account for complex study design. Descriptive statistics are used to describe CVH metrics (ideal, intermediate, and poor). Chi-square tests were used to test for differences in proportion. The significance of linear trends was analysed by using study year as categorical variable in logistic, linear, and ordinal regression analyses depending on the outcome variable. Logistic and linear regressions were used to assess the associations between sociodemographic factors and meeting 5–7 CVH metrics and continuous CVH metrics, overall and stratified by sex. Covariates in the multivariable logistic and linear regression models were age group, sex, educational level, number of adult household members, work and residence status. *P* values < 0.05 were considered significant and missing values were excluded from the analysis.

## Results

### Sample characteristics

Cross-sectional data were analysed from 9712 individuals (20–69 years, mean age = 37.6 years, SD = 12.5 years), 2187 in 2007, 2588 in 2014, and 4,937 in 2019. Further sociodemographic characteristics of the sample by study year are described in Table 1 (see Table 1).

**Table 1:** Sample characteristics of participants aged 20–69 years, Bhutan, 2007, 2014, and 2019.

Variable	Study year		
	2007	2014	2019
	N = 2187	N = 2588^a^	N = 4937^b^
	N (%)	N (%)	N (%)
**Age (years)**			
20–34	520 (23.8)	902 (34.9)	1832 (37.1)
35–49	807 (36.9)	1029 (39.8)	1791 (36.3)
50–69	860 (39.3)	657 (25.4)	1314 (26.6)
**Gender**			
Female	1172 (53.6)	1575 (60.9)	3017 (61.1)
Male	1015 (46.4)	1013 (39.1)	1920 (38.9)
**Education (in years)**			
0	1440 (65.9)	1642 (63.4)	2579 (52.2)
1–10	472 (21.6)	673 (26.0)	1400 (28.4)
≥ 11	273 (12.5)	273 (10.5)	958 (19.4)
**Employment**			
Non-employed	1275 (58.6)	685 (26.5)	3694 (74.8)
Employed	900 (41.4)	1903 (73.5)	1243 (25.2)
**Adult household members**			
0–2	701 (32.2)	1821 (70.4)	1433 (29.0)
3	585 (26.9)	449 (17.4)	1212 (24.5)
4 or more	888 (40.8)	317 (12.3)	2292 (46.4)
**Residence**			
Rural	0 (0.0)	775 (29.9)	3124 (63.3)
Urban	2187 (100)	1813 (70.1)	1813 (36.7)

Unweighted %; ^a^20 (0.8%) were excluded due to history of CVD; ^b^168 (3.3%) were excluded due to history of CVD.

### Distribution of cardiovascular health metrics by study year

Trend analyses showed that ideal physical activity, ideal total cholesterol, ideal blood pressure, and ideal fasting glucose increased from 2007 to 2019, while ideal fruit and vegetable intake, ideal smoking, and ideal body mass index decreased from 2007 to 2019. Five to seven ideal CVH metrics, 0–7 ideal CVH metrics and 0–14 poor, intermediate, and ideal CVH metrics increased from 2007 to 2019. The prevalence of five to seven ideal CVH metrics increased from 11.6% in 2007 to 37.9% in 2019, the seven-mean ideal CVH metrics increased from 2.9 in 2007 to 4.2 in 2019, and the 12-mean ideal CVH metrics increased from 8.7 in 2007 to 10.2 in 2019 (see Table 2).

**Table 2:** Cardiovascular health (CVH) metrics distribution by study year in percent.

Cardiovascular health metrics	Study year			*p* for trend
	2007	2014	2019	
	N = 2187	N = 2588	N = 4937	
**Smoking**				
Poor	7.1	7.4	10.4	**0.015**
Ideal	92.9	92.6	89.6	
**Body mass index**				
Poor	52.9	32.9	47.4	> 0.001
Intermediate	19.2	22.8	18.3	
Ideal	27.9	44.3	34.3	
**Fruit/vegetable intake**				
Poor	14.8	20.1	27.1	> 0.001
Intermediate	45.3	43.6	56.7	
Ideal	39.9	36.3	16.2	
**Physical activity**				
Poor	49.5	6.6	7.1	> 0.001
Intermediate	13.9	5.4	8.2	
Ideal	36.6	88.0	84.7	
**Total cholesterol**				
Poor	9.2	1.8	1.9	> 0.001
Intermediate	25.5	6.2	5.5	
Ideal	65.4	92.0	92.7	
**Blood pressure**				
Poor	19.9	34.0	26.6	> 0.001
Intermediate	44.3	40.4	43.3	
Ideal	35.8	25.6	30.1	
**Fasting glucose**				
Poor	7.1	2.1	1.9	> 0.001
Intermediate	59.7	15.5	6.3	
Ideal	33.2	82.4	91.9	
**No of ideal CVH metrics**				
0	0.6	0.1	0.0	> 0.001
1	12.0	0.4	0.4	
2	27.5	3.4	3.1	
3	29.2	15.5	16.8	
4	19.0	40.7	41.7	
5	9.6	30.8	28.0	
6	2.0	9.0	9.9	
7	0.0	0.0	0.0	
≥ 5	11.6	39.9	37.9	> 0.001
	M (SD)	M (SD)	M (SD)	
0–7	2.9 (1.2)	4.3 (1.0)	4.2 (1.0)	> 0.001
0–14	8.7 (2.2)	10.6 (1.7)	10.2 (1.8)	> 0.001

### Distribution of cardiovascular health metrics by age group and study year

Table 3 shows CVH metrics by age group (20–34 years, 35–49, and 50–69 years). The proportion of ideal smoking increased with higher age group during each study year. The prevalence of ideal BMI decreased by age group during each study year. Ideal fruit and vegetable intake and ideal physical activity did not differ significantly by age group in each study year. The proportion of ideal total cholesterol, ideal blood pressure and ideal fasting glucose decreased with higher age groups in each study year (see Table 3).

**Table 3:** Cardiovascular health (CVH) metrics distribution by age group and study year in percent.

Cardiovascular health metrics	Study year: 2007 N = 2187			Study year: 2014 N = 2626			Study year: 2019 N = 5155		
Age group	20–34	35–49	50–69	20–34	35–49	50–69	20–34	35–49	50–69
**Smoking**									
Poor	11.1	3.9	5.4	11.6	3.9	3.8	13.9	7.6	3.4
Ideal	88.9	96.1	94.6	88.4	96.1	96.2	86.1	92.4	96.6
*p* for trend	> 0.001			> 0.001			> 0.001		
**Body mass index**									
Poor	44.2	57.5	59.7	27.6	37.9	35.0	33.8	57.2	52.1
Intermediate	19.9	21.5	14.7	24.4	23.1	17.5	18.2	17.5	17.4
Ideal	35.9	21.0	25.5	48.0	38.9	47.5	48.0	25.2	30.4
	> 0.001			> 0.001			> 0.001		
**Fruit/vegetable intake**									
Poor	14.3	14.1	16.5	20.1	20.2	19.9	28.8	24.7	31.2
Intermediate	44.5	44.3	48.1	44.2	42.2	45.6	57.3	55.7	55.1
Ideal	41.3	41.6	35.4	35.8	37.5	34.5	13.8	19.5	13.6
	0.063			0.919			0.364		
**Physical activity**									
Poor	51.1	45.5	53.1	7.7	4.9	7.1	6.7	6.9	7.4
Intermediate	13.8	14.0	14.0	6.0	3.9	6.3	10.0	7.3	7.2
Ideal	35.1	40.6	32.9	86.3	91.2	86.5	83.3	85.8	85.4
	0.740			0.392			0.300		
**Total cholesterol**									
Poor	8.2	8.5	11.7	1.0	1.8	3.3	1.0	1.8	3.3
Intermediate	19.4	27.4	32.0	4.0	6.8	9.7	2.7	7.1	8.7
Ideal	72.3	64.1	56.3	95.0	91.4	87.0	96.2	91.2	88.0
	> 0.001			> 0.001			> 0.001		
**Blood pressure**									
Poor	9.2	19.8	37.0	19.6	41.2	53.6	12.3	35.3	43.9
Intermediate	44.5	45.5	42.2	46.1	36.4	33.5	44.7	40.7	42.1
Ideal	46.4	34.6	20.8	34.3	22.4	12.9	43.1	24.0	14.0
	> 0.001			> 0.001			> 0.001		
**Fasting glucose**									
Poor	3.8	6.8	12.9	0.6	2.9	4.2	0.5	2.4	4.0
Intermediate	53.3	62.8	63.8	10.8	19.4	19.0	3.3	7.4	10.7
Ideal	42.9	30.3	23.2	88.6	77.7	76.8	96.2	90.2	85.2
	> 0.001			> 0.001			> 0.001		
**No of ideal CVH metrics**									
0	0.9	0.5	0.5	0.0	0.2	0.0	0.0	0.1	0.0
1	8.6	11.7	17.1	0.3	0.5	0.5	0.3	0.4	0.6
2	20.3	28.9	36.3	2.3	3.7	6.0	1.4	4.1	5.6
3	29.4	30.1	27.9	13.1	16.2	18.3	11.6	18.8	22.3
4	22.8	19.7	12.4	37.9	43.6	41.4	36.1	46.1	41.7
5	14.9	7.2	5.2	33.5	28.0	28.6	33.3	23.7	24.6
6	3.1	2.0	0.5	12.9	7.8	5.2	17.4	6.8	5.2
7	0.0	0.0	0.0	0.0	0.0	0.0	0.0	0.0	0.0
≥ 5	18.0	9.2	5.9	46.4	35.8	33.7	50.7	30.5	29.8
	M (SD)	M (SD)	M (SD)	M (SD)	M (SD)	M (SD)	M (SD)	M (SD)	M (SD)
0–7	3.2 (1.3)	2.9 (1.2)	2.5 (1.1)	4.4 (1.0)	4.2 (1.0)	4.1 (1.0)	4.4 (1.0)	4.1 (1.0)	4.0 (1.0)
0–14	9.2 (2.2)	8.7 (2.1)	7.9 (2.1)	10.9 (1.7)	10.4 (1.7)	10.1 (1.8)	10.5 (1.7)	9.9 (1.8)	9.7 (1.9)

### Distribution of cardiovascular health metrics by sex and study year

Table 4 shows CVH metrics by sex. Ideal smoking, and ideal blood pressure were higher among women than among men in all study years, while ideal BMI and ideal physical activity were higher among men than women, and ideal fruit and vegetable consumption, ideal FBG and ideal total cholesterol did not have clear sex differences (see Table 4).

**Table 4:** Cardiovascular health (CVH) metrics distribution by sex and study year in percent.

Cardiovascular health metrics	Study year: 2007 N = 2187		Study year: 2014 N = 2626		Study year: 2019 N = 5155	
	Male	Female	Male	Female	Male	Female
**Smoking**						
Poor	8.7	5.0	10.3	3.4	15.7	3.8
Ideal	91.3	95.0	89.7	96.6	84.3	96.2
*p*-value	0.002		< 0.001		< 0.001	
**Body mass index**						
Poor	52.0	54.1	26.9	41.1	42.7	55.3
Intermediate	20.7	17.5	24.7	20.2	19.1	17.2
Ideal	27.4	28.4	48.4	38.6	38.2	29.5
*p*-value	0.207		< 0.001		< 0.001	
**Fruit/vegetable intake**						
Poor	13.9	15.9	18.6	22.2	25.8	28.6
Intermediate	43.7	47.4	42.8	44.7	57.7	55.5
Ideal	42.4	36.8	38.6	33.1	16.5	15.9
*p*-value	0.040		0.130		0.257	
**Physical activity**						
Poor	40.8	60.6	4.1	9.9	6.4	8.0
Intermediate	14.2	13.5	3.9	7.3	6.0	10.9
Ideal	45.0	25.9	92.0	82.8	87.6	81.0
*p*-value	< 0.001		< 0.001		< 0.001	
**Total cholesterol**						
Poor	10.5	7.5	1.4	2.2	1.7	2.1
Intermediate	25.5	25.3	6.4	5.9	5.0	6.0
Ideal	63.9	67.2	92.2	91.9	93.3	91.9
*p*-value	0.049		0.441		0.360	
**Blood pressure**						
Poor	22.2	17.0	32.1	35.2	31.1	21.0
Intermediate	48.1	39.4	44.7	34.7	45.6	40.4
Ideal	29.7	43.6	22.3	30.1	23.3	38.6
*p*-value	< 0.001		< 0.001		< 0.001	
**Fasting glucose**						
Poor	7.0	7.3	2.3	2.0	1.9	1.8
Intermediate	59.2	60.3	15.7	15.1	6.6	5.8
Ideal	33.8	32.4	82.0	82.9	91.5	92.4
*p*-value	0.804		0.896		0.696	
**No of ideal CVH metrics**						
0	0.9	0.4	0.1	0.0	0.1	0.0
1	12.9	10.5	0.5	0.4	0.6	0.3
2	26.9	27.9	3.1	3.9	3.4	2.8
3	28.7	30.0	14.7	16.6	18.6	14.7
4	17.4	21.3	41.5	39.7	40.9	42.6
5	11.1	7.9	31.3	30.1	27.6	28.5
6	2.1	2.0	8.9	9.2	8.9	11.1
7	0.0	0.0	0.0	0.0	0.0	0.0
≥ 5	13.3	9.8	40.2	39.3	36.5	39.6
	M (SD)	M (SD)	M (SD)	M (SD)	M (SD)	M (SD)
0–7	2.9 (1.3)	2.9 (1.2)	4.3 (1.0)	4.2 (1.0)	4.2 (1.0)	4.3 (1.0)
0–14	8.8 (2.3)	8.6 (2.1)	10.7 (1.8)	10.4 (1.8)	10.1 (1.8)	10.3 (1.8)

### Associations with meeting 5–7 ideal CVH metrics

In adjusted logistic regression analysis, older age was negatively associated with meeting 5–7 ideal CVH metrics in all three study years. Male sex increased the odds, and employment decreased the odds of 5–7 ideal CVH metrics in 2007, and urban residence increased the odds of 5–7 ideal CVH metrics in 2014 and decreased the odds in 2019. Furthermore, in the sex stratified analysis, higher education increased the odds of 5–7 ideal CVH metrics in 2019 among women (see Table 5).

**Table 5:** Adjusted associations with meeting 5–7 ideal cardiovascular health metrics.

Variable	Study year		
	2007	2014	2019
	Adjusted OR (95% CI)	Adjusted OR (95% CI)	Adjusted OR (95% CI)
*Total sample*			
**Age (years)**			
20–34	1 (Reference)	1 (Reference)	1 (Reference)
35–49	0.46 (0.33, 0.66)***	0.63 (0.50, 0.81)***	0.54 (0.43, 0.68)***
50–69	0.29 (0.19, 0.43)***	0.52 (0.39, 0.68)***	0.51 (0.38, 0.69)***
**Gender**			
Female	1 (Reference)	1 (Reference)	1 (Reference)
Male	1.71 (1.18, 2.46)**	1.05 (0.83, 1.33)	0.89 (0.74, 1.07)
**Education (in years)**			
0	1 (Reference)	1 (Reference)	1 (Reference)
1–10	1.05 (0.72, 1.55)	0.95 (0.71, 1.27)	1.18 (0.99, 1.41)
≥ 11	0.85 (0.51, 1.44)	1.09 (0.72, 1.65)	1.35 (1.00, 1.83)
**Employment**			
Non-employed	1 (Reference)	1 (Reference)	1 (Reference)
Employed	0.63 (0.42, 0.92)*	0.90 (0.71, 1.14)	0.83 (0.66, 1.05)
**Adult household members**			
0–2	1 (Reference)	1 (Reference)	1 (Reference)
3	0.75 (0.50, 1.11)	1.27 (0.95, 1.70)	1.06 (0.83, 1.35)
4 or more	0.73 (0.50, 1.07)	1.10 (0.79, 1.52)	1.03 (0.84, 1.25)
**Residence**			
Rural	–	1 (Reference)	1 (Reference)
Urban		1.95 (1.44, 2.63)***	0.66 (0.54, 0.82)***
*Male*			
**Age (years)**			
20–34	1 (Reference)	1 (Reference)	1 (Reference)
35–49	0.42 (0.26, 0.68)***	0.70 (0.47, 1.04)	0.57 (0.40, 0.81)**
50–69	0.23 (0.13, 0.41)***	0.55 (0.37, 0.80)**	0.56 (0.38, 0.85)**
**Education (in years)**			
0	1 (Reference)	1 (Reference)	1 (Reference)
1–10	1.01 (0.61, 1.65)	0.88 (0.58, 1.34)	1.16 (0.85, 1.58)
≥ 11	0.68 (0.36, 1.40)	0.85 (0.47, 1.53)	1.04 (0.67, 1.62)
**Employment**			
Non-employed	1 (Reference)	1 (Reference)	1 (Reference)
Employed	0.47 (0.30, 0.77)***	0.82 (0.50, 1.33)	0.75 (0.54, 1.06)
**Adult household members**			
0–2	1 (Reference)	1 (Reference)	1 (Reference)
3	0.75 (0.43, 1.29)	1.39 (0.86, 2.17)	0.99 (0.70, 1.41)
4 or more	0.68 (0.40, 1.15)	0.93 (0.59, 1.48)	0.94 (0.69, 1.29)
**Residence**			
Rural	–	1 (Reference)	1 (Reference)
Urban		1.98 (1.27, 3.08)**	0.65 (0.48, 0.88)**
*Female*			
**Age (years)**			
20–34	1 (Reference)	1 (Reference)	1 (Reference)
35–49	0.57 (0.34, 0.96)*	0.55 (0.42, 0.72)***	0.53 (0.41, 0.69)***
50–69	0.41 (0.23, 0.73)***	0.47 (0.33, 0.68)***	0.44 (0.32, 0.61)***
**Education (in years)**			
0	1 (Reference)	1 (Reference)	1 (Reference)
1–10	1.01 (0.55, 1.86)	1.04 (0.76, 1.42)	1.12 (0.86, 1.46)
≥ 11	1.31 (0.55, 3.12)	1.66 (0.95, 2.90)	1.93 (1.36, 2.75)***
**Employment**			
Non-employed	1 (Reference)	1 (Reference)	1 (Reference)
Employed	1.08 (0.60, 1.95)	0.99 (0.73, 1.33)	0.96 (0.70, 1.32)
**Adult household members**			
0–2	1 (Reference)	1 (Reference)	1 (Reference)
3	0.77 (0.43, 1.37)	1.19 (0.86, 1.66)	1.16 (0.88, 1.53)
4 or more	0.74 (0.43, 1.28	1.40 (0.93, 2.10)	1.17 (0.91, 1.50)
**Residence**			
Rural	–	1 (Reference)	1 (Reference)
Urban		1.90 (1.30, 2.78)***	0.69 (0.53, 0.89)**

OR = odds ratio; CI = confidence intervals.

*p < 0.05; **p < 0.01; ***p < 0.001.

### Associations with continuous CVH metrics

In the adjusted linear regression analysis, older age was negatively associated with continuous CVH metrics in the three study years. Male sex was positively associated with continuous CVH metrics in 2007 and 2014, higher education was positively associated with continuous CVH metrics in 2019 and being employed was in 2019, and a higher number of adult household members was in 2007 negatively associated with continuous CVH metrics. Urban residence was in 2014 positively and in 2019 negatively associated with continuous CVH metrics. Similar results were found in sex stratified analysis (see Table 6).

**Table 6:** Adjusted associations with ideal cardiovascular health metrics (0–14).

Variable	Study year		
	2007	2014	2019
	Adjusted Exp (Coef) (95% CI)	Adjusted Exp (Coef) (95% CI)	Adjusted Exp (Coef) (95% CI)
*Total sample*			
**Age (years)**			
20–34	1 (Reference)	1 (Reference)	1 (Reference)
35–49	0.60 (0.47, 0.77)**	0.61 (0.51, 0.73)***	0.56 (0.45, 0.70)***
50–69	0.27 (0.21, 0.35)***	1.12 (0.78, 1.60)	0.41 (0.32, 0.53)***
**Gender**			
Female	1 (Reference)	1 (Reference)	1 (Reference)
Male	1.32 (1.06, 1.64)*	1.28 (1.05, 1.57)*	0.87 (0.75, 1.01)
**Education (in years)**			
0	1 (Reference)	1 (Reference)	1 (Reference)
1–10	0.91 (0.71, 1.17)	0.94 (0.73, 1.20)	1.06 (0.91, 1.24)
≥ 11	0.74 (0.53, 1.04)	1.12 (0.78, 1.60)	1.30 (1.04, 1.61)*
**Employment**			
Non-employed	1 (Reference)	1 (Reference)	1 (Reference)
Employed	0.82 (0.65, 1.03)	1.03 (0.80, 1.33)	0.81 (0.67, 0.98)*
**Adult household members**			
0–2	1 (Reference)	1 (Reference)	1 (Reference)
3	0.74 (0.56, 0.96)*	1.07 (0.83, 1.37)	1.14 (0.94, 1.39)
4 or more	0.93 (0.57, 0.94)*	0.97 (0.75, 1.26)	1.05 (0.89, 1.24)
**Residence**			
Rural	–	1 (Reference)	1 (Reference)
Urban		2.10 (1.60, 2.75)***	0.58 (0.46, 0.74)***
*Male*			
**Age (years)**			
20–34	1 (Reference)	1 (Reference)	1 (Reference)
35–49	0.59 (0.41, 0.85)**	0.65 (0.50, 0.84)***	0.59 (0.43, 0.81)***
50–69	0.27 (0.19, 0.40)***	0.46 (0.34, 0.62)***	0.50 (0.35, 0.72)***
**Education (in years)**			
0	1 (Reference)	1 (Reference)	1 (Reference)
1–10	0.87 (0.62, 1.24)	0.79 (0.57, 1.10)	0.92 (0.71, 1.21)
≥ 11	0.58 (0.38, 0.88)**	0.89 (0.53, 1.49)	0.95 (0.69, 1.32)
**Employment**			
Non-employed	1 (Reference)	1 (Reference)	1 (Reference)
Employed	0.63 (0.46, 0.86)**	0.93 (0.61, 1.42)	0.78 (0.57, 1.06)
**Adult household members**			
0–2	1 (Reference)	1 (Reference)	1 (Reference)
3	0.68 (0.46, 1.00)*	1.05 (0.75, 1.47)	1.23 (0.93, 1.63)
4 or more	0.71 (0.49, 1.02)	0.91 (0.63, 1.31)	1.09 (0.84, 1.41)
**Residence**			
Rural	–	1 (Reference)	1 (Reference)
Urban		2.40 (1.62, 3.55)***	0.54 (0.39, 0.75)***
*Female*			
**Age (years)**			
20–34	1 (Reference)	1 (Reference)	1 (Reference)
35–49	0.67 (0.49, 0.91)*	0.61 (0.47, 0.79)***	0.56 (0.47, 0.68)***
50–69	0.29 (0.21, 0.40)***	0.38 (0.29, 0.51)***	0.34 (0.26, 0.43)***
**Education (in years)**			
0	1 (Reference)	1 (Reference)	1 (Reference)
1–10	0.88 (0.61, 1.26)	1.22 (0.88, 1.70)	1.15 (0.94, 1.42)
≥ 11	1.41 (0.83, 2.41)	1.77 (1.12, 2.78)*	1.99 (1.57, 2.52)***
**Employment**			
Non-employed	1 (Reference)	1 (Reference)	1 (Reference)
Employed	1.15 (0.84, 1.56)	1.29 (0.98, 1.69)	0.89 (0.70, 1.13)
**Adult household members**			
0–2	1 (Reference)	1 (Reference)	1 (Reference)
3	0.83 (0.59, 1.16)	1.21 (0.91, 1.62)	1.03 (0.85, 1.27)
4 or more	0.73 (0.54, 1.00)*	1.18 (0.88, 1.60)	1.02 (0.83, 1.26)
**Residence**			
Rural	–	1 (Reference)	1 (Reference)
Urban		1.73 (1.23, 2.45)**	0.64 (0.51, 0.82)***

Exp (Coef) = Exponentiated Coefficient CI = Confidence Intervals.

*p < 0.05; **p < 0.01; ***p < 0.001.

## Discussion

The researchers found that the prevalence of ideal CVH (5–7 ideal metrics) in Bhutan increased from 11.6% in 2007 to 37.9% in 2019, and the mean CVH metrics increased from 2.9 in 2007 to 4.2 in 2019. A similar increase in the ideal CVH score was found in a trend study in the USA (increase from 3.89 in 2011 to 3.96 in 2017^20^, while the mean CVH metrics increased in Iran from 4.7 in 2007 to 5.0 in 2016^19^. The increase in ideal CVH metrics in this study was mainly attributed to ideal physical activity, ideal total cholesterol, ideal blood pressure, and ideal blood glucose. However, we saw decreases in the proportion of ideal fruit and vegetable, ideal smoking, and ideal body mass index from 2007 to 2019.

The prevalence of ideal CVH (5–7 ideal metrics) (ranging from 11.6% in 2007 to 37.9% in 2019), were similar to global estimates, mainly in high-income countries, ideal CVH (having 5–7 ideal metrics) (19.6%) CVH^8^, and in China (33.0%, 5–7 ideal metrics)^15^, but lower than in Nepal (51.6%, 5–7 ideal metrics)^16^. The proportion of ideal CVH metrics (all 7 metrics) (0.6% in 2007, 0.1% in 2014, and 0.0% in 2019), was similar to in previous studies, such as in urban India (< 0.1% had 7 ideal metrics)^17^, Shandon in China (0.05% all 7 ideal metrics)^12^, in rural area Northwest China all 7 ideal metrics (0.0%)^13^.

Like the three best global estimates^8^, this survey found that TC (92.7% in 2019), smoking (89.6% in 2019), and FGP (91.9% in 2019) had the highest proportion of ideal CVH status. A healthy diet (16.2% in 2019) had the poorest prevalence of ideal CVH status in this study which compares with the poorest global estimates^8^. The proportion of ideal fruit and vegetable consumption compares with global rates in low- and middle-income countries (18.0%)^33^. The estimates of ideal PA (84.7% in 2019) in this study were higher than global estimates of PA (40.6%) and ideal BMI (34.3% in 2019) are lower than global figures of ideal BMI (40.3%)^8^. Ideal blood pressure significantly decreased from 35.8% in 2007 to 30.1% in 2019 and poor blood pressure or hypertension significantly increased from 19.9% in 2007 to 26.6% in 2019, which is higher than global estimates in low- and middle-income countries (17.5%)^34^. Previous research in Bhutan also found a high prevalence of poor diet, overweight, and high blood pressure among adults calling for expanded supplementation and dietary interventions^3^.

Consistent with previous research^8,16,17,22,23^, ideal CVH was higher among younger age groups (20–34 years) and among men in 2007. Some previous research^12,22,23^ showed an association between higher education and ideal CVH metrics, while we only found this result, in particular among women, in 2019. Other research^24,25^, found an association between rural residence and ideal CVH metrics, which was also found in this study in 2019, while we found higher ideal CVH metrics in residents of urban than rural areas in 2014.

Results may inform Bhutan’s CVH promotion policy. The increase in ideal physical activity may be attributed to the “implementation of community wide public education and awareness campaigns for physical activity”, including a nationwide “Move for Health Campaign” is conducted routinely and open-air gyms in Thimphu and a number of other districts^3,35^. Diabetes and raised total cholesterol may have reduced due to the universal health coverage, including the early diagnosis and management of NCDs in Bhutan^3^. To improve CVH in Bhutan, multifactorial interventions are needed^36^, targeting the promotion of body weight control, smoking cessation, healthy diets, and screening and control of high levels of blood pressure^5^.

In addition to the use of large representative survey samples and standardized assessment measures, the study limitations included the cross-sectional design and self-report of some of the data. In addition, our healthy diet measure only consisted of one item (fruit and vegetable consumption) and not as in the original AHA healthy diet definition “(≥ 4.5 cups/day fruits and vegetables, 2: ≥ 3.5-oz servings/week of fish, 3: < 1500 mg/day sodium, 4: < 450 cal/week sweets/sugar, and 5: ≥ 3 1-oz servings/day whole grains)”^8,9^. Another limitation was that the history of any previous CVD was not evaluated in the 2007 and only in the 2014 and 2019 survey.

## Conclusion

The proportion of meeting 5–7 ideal CVH metrics increased in Bhutan. Primary and secondary prevention programmes should be strengthened to improve CVH in Bhutan, considering identified associated factors. Future research may include more comprehensive measures on a healthy diet.

## Data Availability

The data source is publicly available at the World Health Organization NCD Microdata Repository (URL: https://extranet.who.int/ncdsmicrodata/index.php/catalog).

## References

[1] World Health Organization (WHO). Cardiovascular diseases (CVDs), 2021. https://www.who.int/news-room/fact-sheets/detail/cardiovascular-diseases-(cvds). Accessed 11 January 2022

[2] World Health Organization (WHO) Bhutan: Noncommunicable Diseases (NCD) Country Profiles, 2018. https://www.who.int/nmh/countries/btn_en.pdf?ua=1. Accessed 2 Aug 2021.

[3] United Nations Interagency Task Force on the Prevention and Control of Noncommunicable Diseases. Joint Mission, Bhutan, 6–10 February 2017. Geneva: World Health Organization; 2017 (WHO/NMH/NMA/17.57). Licence: CC BY-NC-SA 3.0 IGO.

[4] D'Agostino RB, Vasan RS, Pencina MJ, Wolf PA, Cobain M, Massaro JM, Kannel WB (2008). General cardiovascular risk profile for use in primary care: The Framingham Heart Study. Circulation.

[5] Gyawali B, Mishra SR, Virani SS (2019). Low levels of ideal cardiovascular health in a semi-urban population of Western Nepal: A population-based, cross-sectional study. Heart Asia.

[6] Lloyd-Jones DM, Hong Y, Labarthe D (2010). Defining and setting national goals for cardiovascular health promotion and disease reduction: The American Heart Association's strategic Impact Goal through 2020 and beyond. Circulation.

[7] Huffman MD, Capewell S, Ning H (2012). Cardiovascular health behavior and health factor changes (1988–2008) and projections to 2020: Results from the National Health and Nutrition Examination Surveys. Circulation.

[8] Peng Y, Cao S, Yao Z (2018). Prevalence of the cardiovascular health status in adults: A systematic review and meta-analysis. Nutr. Metab. Cardiovasc. Dis..

[9] Younus A, Aneni EC, Spatz ES (2016). A systematic review of the prevalence and outcomes of ideal cardiovascular health in US and non-US populations. Mayo Clin. Proc..

[10] Isiozor NM, Kunutsor SK, Voutilainen A, Kurl S, Kauhanen J, Laukkanen JA (2019). American heart association's cardiovascular health metrics and risk of cardiovascular disease mortality among a middle-aged male Scandinavian population. Ann. Med..

[11] Gao B, Wang F, Zhu M, Wang J, Zhou M, Zhang L, Zhao M (2020). Cardiovascular health metrics and all-cause mortality and mortality from major non-communicable chronic diseases among Chinese adult population. Int. J. Cardiol..

[12] Ren J, Guo XL, Lu ZL (2016). Ideal cardiovascular health status and its association with socioeconomic factors in Chinese adults in Shandong, China. BMC Public Health.

[13] Zhao Y, Yan H, Yang R (2016). Status of cardiovascular health among adults in a rural area of Northwest China: Results from a cross-sectional study. Medicine (Baltimore).

[14] Chang Y, Guo X, Chen Y (2016). Prevalence and metrics distribution of ideal cardiovascular health: A population-based, cross-sectional study in rural China. Heart Lung Circ..

[15] Bi Y, Jiang Y, He J (2015). Status of cardiovascular health in Chinese adults. J. Am. Coll. Cardiol..

[16] Ghimire U, Shrestha N, Gyawali B (2020). Prevalence of American Heart Association defined ideal cardiovascular health metrics in Nepal: Findings from a nationally representative cross-sectional study. Int. Health..

[17] Gupta B, Gupta R, Sharma KK (2017). Low prevalence of AHA-defined ideal cardiovascular health factors: A study of urban Indian men and women. Glob. Heart..

[18] Yu Y, Dong Z, Li Y, Zhang J, Yin S, Gao X, Wu S (2021). The Cardiovascular and Cerebrovascular Health in North China From 2006 to 2011: Results From the KaiLuan Study. Front. Cardiovasc. Med..

[19] Rahmani F, Asgari S, Khalili D, Habibi Moeini AS, Tohidi M, Azizi F, Hadaegh F (2021). National trends in cardiovascular health metrics among Iranian adults using results of three cross-sectional STEPwise approaches to surveillance surveys. Sci. Rep..

[20] Parcha V, Kalra R, Suri SS, Malla G, Wang TJ, Arora G, Arora P (2021). Geographic variation in cardiovascular health among American adults. Mayo Clin. Proc..

[21] Gaye B, Tajeu GS, Offredo L, Vignac M, Johnson S, Thomas F, Jouven X (2020). Temporal trends of cardiovascular health factors among 366 270 French adults. Eur. Heart J. Qual. Care Clin. Outcomes..

[22] Machado LBM, Silva BLS, Garcia AP (2018). Ideal cardiovascular health score at the ELSA-Brazil baseline and its association with sociodemographic characteristics. Int. J. Cardiol..

[23] Magodoro IM, Feng M, North CM (2019). Female sex and cardiovascular disease risk in rural Uganda: A cross-sectional, population-based study. BMC Cardiovasc. Disord..

[24] van Nieuwenhuizen B, Zafarmand MH, Beune E (2018). Ideal cardiovascular health among Ghanaian populations in three European countries and rural and urban Ghana: The RODAM study. Intern. Emerg. Med..

[25] Benziger CP, Zavala-Loayza JA, Bernabe-Ortiz A (2018). Low prevalence of ideal cardiovascular health in Peru. Heart.

[26] World Health Organization (WHO) (2018) STEPwise approach to surveillance (STEPS). https://www.who.int/ncds/surveillance/steps/en/. Accessed 22 August 2021

[27] Ministry of Health 2009. Report on 2007 Steps Survey for Risk Factors and Prevalence of Noncommunicable Diseases in Thimphu. https://extranet.who.int/ncdsmicrodata/index.php/catalog/738/related-materials. Accessed 5 August 2021

[28] World Health Organization, Regional Office for South-East Asia. National survey for noncommunicable disease risk factors and mental health using WHO STEPS approach in Bhutan, 2014. https://extranet.who.int/ncdsmicrodata/index.php/catalog/427/related-materials. Accessed 5 August 2021

[29] Department of Public Health, Ministry of Health (2020). Non-communicable disease Risk Factors: Bhutan STEPS Survey 2019, Thimphu. https://extranet.who.int/ncdsmicrodata/index.php/catalog/855/related-materials. Accessed 5 August 2021

[30] Wen CP, David Cheng TY, Tsai SP, Chan HT, Hsu HL, Hsu CC, Eriksen MP (2009). Are Asians at greater mortality risks for being overweight than Caucasians? Redefining obesity for Asians. Public Health Nutr..

[31] Pengpid S, Peltzer K (2021). Ideal cardiovascular health in a nationally representative population-based sample of adults in Malawi. Glob. Heart..

[32] Armstrong T, Bull F (2006). Development of the World Health Organization Global Physical Activity Questionnaire (GPAQ). J. Public Health.

[33] Frank SM, Webster J, McKenzie B (2019). Consumption of fruits and vegetables among individuals 15 years and older in 28 low- and middle-income countries. J. Nutr..

[34] Geldsetzer P, Manne-Goehler J, Marcus ME (2019). The state of hypertension care in 44 low-income and middle-income countries:Aa cross-sectional study of nationally representative individual-level data from 1·million adults. Lancet.

[35] Tuangratananon T, Wangmo S, Widanapathirana N, Pongutta S, Viriyathorn S, Patcharanarumol W (2019). Implementation of national action plans on noncommunicable diseases, Bhutan, Cambodia, Indonesia, Philippines, Sri Lanka, Thailand and Viet Nam. Bull. World Health Organ..

[36] Moghaddam MM, Mohebi R, Hosseini F (2014). Distribution of ideal cardiovascular health in a community-based cohort of Middle East population. Ann. Saudi Med..

